# Association of *FGF4L1* Retrogene Insertion with Prolapsed Gland of the Nictitans (Cherry Eye) in Dogs

**DOI:** 10.3390/genes15020198

**Published:** 2024-02-01

**Authors:** Jamie Freyer, Julia D. Labadie, Jason T. Huff, Michael Denyer, Oliver P. Forman, Rebecca Chodroff Foran, Jonas Donner

**Affiliations:** 1 Wisdom Panel, Mars Petcare Science and Diagnostics, Portland, OR 97209, USA; jdlabadie@gmail.com (J.D.L.); jason.huff@wisdompanel.com (J.T.H.); becca.foran@wisdompanel.com (R.C.F.); 2 Wisdom Panel, Mars Petcare Science and Diagnostics, Waltham on the Wolds, Leicestershire LE14 4RS, UK; michael.denyer@wisdompanel.com (M.D.); oliver.forman@wisdompanel.com (O.P.F.); 3 Wisdom Panel, Mars Petcare Science and Diagnostics, 00581 Helsinki, Finland; jonas.donner@wisdompanel.com

**Keywords:** cherry eye, prolapsed nictitans, third eyelid gland, canine, GWAS, genetics, *FGF4*, *FGF4L1*, chondrodysplasia

## Abstract

Cherry eye is the common name for prolapse of the nictitans gland, a tear-producing gland situated under the third eyelid of dogs. Cherry eye is characterized by a red fleshy protuberance in the corner of the eye, resembling a cherry. This protrusion is a displacement of the normal gland of the third eyelid, thought to be caused by a defect in the connective tissue that secures the gland in place. Options for treatment may include anti-inflammatory medications in mild cases, but surgical replacement of the gland is usually indicated. Cherry eye is most often seen in dogs under the age of two years, with certain breeds having a higher incidence, suggesting a potential genetic association. Integration of panel genetic testing into routine clinical practice allows for the generation of large numbers of genotyped individuals paired with clinical records and enables the investigation of common disorders using a genome-wide association study (GWAS) approach at scale. In this investigation, several thousand cases and controls for cherry eye in both purebred dogs and mixed breeds are used for a large-scale GWAS, revealing a single peak of genome-wide significance on canine chromosome 18, directly at the location of the previously identified *FGF4* insertion known to cause chondrodysplasia in several breeds.

## 1. Introduction

The canine third eyelid, or nictitans, is a crescent-shaped conjunctival fold in the ventromedial aspect of the eye, conforming to the shape of the cornea and confluent with the conjunctival mucosa. Structural support for the eyelid is provided by a T-shaped wedge of hyaline cartilage [1,2]. The nictitans provides barrier protection to the globe, removes ocular debris, helps distribute tears, contributes mucin to the preocular film, and houses the third eyelid gland [3]. This gland is a tubuloacinar, seromucoid gland that is estimated to produce 30–60% of the total aqueous volume of tears [4].

Prolapsed gland of the nictitans, colloquially known as “cherry eye”, is the most common disorder of the third eyelid in dogs [5] and occurs when the gland of the third eyelid protrudes from the ventral palpebral opening. While the exact etiology is still unknown, it is hypothesized that it stems from a laxity in the connective tissue holding the nictitans in place, resulting in a predisposition toward prolapse [5,6]. Antigen-stimulated enlargement of the gland (lymphoid hyperplasia) due to exposure to environmental allergens in young animals has also been thought to play a possible role [6]. Cherry eye is believed to have a genetic component, with some breeds known to be at increased risk. Studies have shown that the breeds with the highest risk include the Neapolitan Mastiff, English and French Bulldogs, Cane Corso, Lhasa Apso, and American Cocker Spaniel [4,5,7]. Brachycephalic breeds are also overrepresented [8]. Cherry eye is generally a disorder of young dogs, with a mean age at diagnosis of 0.63 years [4] and the majority of cases occurring before 2 years of age [4,5]. It may be unilateral or bilateral, with a 3:2 ratio between the two, respectively [6].

Cherry eye is not commonly painful and does not often damage a dog’s sight but may result in irritation and inflammation if not treated [6]. Additionally, because the third eyelid gland contributes so heavily to tear volume, a lack of treatment for prolapse can cause keratoconjunctivitis sicca (KCS, or “dry eye”) [1]. Surgical treatment is the gold standard of care, as approximately 43% of untreated cases will develop dry eye [9]. Surgical removal of the gland was once considered to be the preferred treatment, but this procedure has fallen out of favor as studies show that approximately 50% of cases in which the gland has been removed develop KCS, compared to 14% in eyes treated with one of the replacement techniques [1,5,7,9,10]. These include several tacking or anchoring procedures, fixing the nictitans to periorbital structures such as the oblique muscles or periorbital fascia, as well as techniques in which a pocket is created in the adjacent mucosa and the gland replaced into the pocket [4,10]. While pocket procedures can do damage to excretory ducts, tacking procedures can reduce the mobility of the nictitans [10]. Recurrence can be an issue with any technique [10], though it has been suggested that tacking procedures may be preferable in cases of chronic or extensive protrusions, while pocket techniques may be recommended for younger animals and milder cases [10].

Because a genetic component is likely, nictitans prolapse generally causes affected individuals to be removed from breeding programs. Cherry eye can also be quite alarming to pet owners, as well as frustrating due to the possibility of recurrence and dry eye, even with treatment. For these reasons, it would be beneficial to understand more about the genetic factors underlying this disorder. In this study, a combination of genetic panel testing and paired electronic medical records was leveraged to obtain the largest affected cohort to date for evaluation of the genetic basis of cherry eye. The present investigation used a large-scale multi-breed genome-wide association study (GWAS) approach to explore the genetics of cherry eye in the dog, using DNA samples collected by veterinarians at Banfield Pet Hospital^®^ locations across the United States and Mexico and through direct-to-consumer DNA testing in the United States.

## 2. Materials and Methods

### 2.1. Samples

DNA samples were collected via commercial testing of Wisdom Panel^TM^ Premium, Wisdom Panel^TM^ Essential, Wisdom Panel^TM^ Health and Optimal Selection^TM^ retail products, and genetic testing was performed as a part of Optimal Wellness Plans^®^ for puppies through Banfield Pet Hospital^®^ branches (Vancouver, WA, USA). Samples were collected through either non-invasive buccal swabbing by dog owners or veterinary professionals or through blood sampling by a veterinary professional at a Banfield Pet Hospital^®^ in line with regulations governing diagnostic testing. Consent for the use of DNA data in research was obtained through the client’s agreement with the terms and conditions of DNA testing through Wisdom Panel. All samples originated in the United States or Mexico.

### 2.2. Genotyping

DNA extraction from whole blood and buccal swabs was performed at GeneSeek Laboratories (Neogen Co., Ltd., Lincoln, NE, USA). Genotyping was performed following manufacturer-suggested standard protocols on a custom 100 k Illumina Infinium XT SNP (single nucleotide polymorphism) microarray (Illumina, Inc., San Diego, CA, USA), also at GeneSeek Laboratories. The microarray contained disease and trait tests, as well as coverage throughout the genome, and was designed and validated for use following the same protocol and principles as previously described [11]. Microarray genotyping analyses were carried out following manufacturer-recommended standard protocols for the Illumina XT platform (Illumina, Inc., San Diego, CA, USA). Only samples achieving at least 97% genotyping call rates were included in the study.

### 2.3. Clinical Information

For DNA samples submitted directly for genotyping through Banfield clinics, data from genotyped dogs were directly linked with clinical records stored in the Banfield electronic medical record (EMR). For DNA samples collected and submitted by general retail consumers of Wisdom Panel products, data from genotyped dogs were linked with the Banfield EMR by anonymized cross-matching of pet and owner information, in accordance with personally identifiable information (PII) regulations. The EMR was then queried for dogs diagnosed with prolapse of one or both nictitating membranes.

### 2.4. Inclusion Criteria for Genetic Analysis

For the ascertainment of a GWAS study population, a total of 7278 dogs were identified with DNA data and a diagnosis of third eyelid gland prolapse with a minimum age of 6 weeks. Controls were defined as dogs not recorded as having a third eyelid gland prolapse in the Banfield EMR that were over the age of 3 years at the time of study and were randomly chosen from a larger subset meeting these criteria. Breed assignment was based on comparison to a reference panel of over 21,000 dogs of known ancestry from more than 50 countries and ascertained using the BCSYS Local Ancestry Classifier algorithm [12]. Dogs were considered to be purebred if determined by the BCSYS algorithm to have 90% or greater single-origin ancestry. For the purposes of breed-specific analyses, the population was subsequently expanded to include dogs with 80% or greater single-origin ancestry in order to obtain cohorts of a reasonable size for analysis.

### 2.5. Genotype Analysis

A total of 95,165 variants were available for analysis. After filtering and removal of samples with greater than 5% missing data, SNPs with a minor allele frequency ≤1%, variants with greater than 5% missing data, and SNPs with an absolute difference in call rate between males and females > 2.5%, a total set of 91,770 variants remained. Genome-wide association study analysis was performed using a linear mixed-model approach performed using the software package GEMMA v0.98.5 [11], including a centered relatedness matrix. All GWAS analyses were performed within the Databricks cloud-based data analytics system. Manhattan and QQ plots were created using the R package qqman v0.1.9 [13], and PCA plots were created using the R package ggplot2 v3.4.3 [14]. All reported genome locations are given based on the CanFam3.1 genome build.

### 2.6. Additional Statistical Analyses

Epidemiological odds ratios (ORs) with 95% confidence intervals in the Banfield EMR cohort of all 769,337 dogs with Banfield data and genetic data were calculated through univariable logistic regression analysis in the Minitab statistical analysis software v21.4. The case and breed definitions used for this analysis were the same as those noted above. The skull shape variable was created by assigning each individual the expected breed-average phenotype (brachycephalic, mesocephalic or dolichocephalic) based on the breed standard. The significance of the *FGF4L1* gene variant frequency distribution between groups of dogs was evaluated using either a chi square test or Fisher’s exact test whenever any expected cell frequency was <5. Spearman’s correlation coefficient was calculated in Minitabto evaluate the correlation between *FGF4L1*-derived allele frequency and the clinical prevalence of cherry eye.

### 2.7. Ethics Statements

Genetic analyses were carried out on DNA extracted from owner-collected, non-invasive cheek swab samples or from blood/cheek swab samples collected at certified veterinary clinics in accordance with international standards for animal care and research. All dog owners provided consent for the use of their dog’s DNA sample for research purposes. As this study was purely based on data analytics, animal research ethics approval was not required.

## 3. Results

### 3.1. Epidemiology

Queries of EMR data for an initial investigation of the epidemiology of cherry eye in a United States primary veterinary care setting returned information for 769,337 dogs, of which 6753 (0.88%) had been diagnosed with cherry eye. Descriptive statistics for the identified case cohort are shown in Table 1. Male dogs were somewhat overrepresented, and neutered/spayed dogs were underrepresented, among cases (OR = 1.10 and OR = 0.79, respectively; Table 1). Purebred dogs (OR = 1.79 [95% confidence interval, CI 1.70–1.88]) were at higher risk for cherry eye compared to mixed-breed dogs. Dogs of breeds with a brachycephalic skull shape phenotype were at particularly high risk of the condition (OR = 7.60 [6.90–8.36]) compared to mesocephalic breeds (Table 1). The vast majority of dogs had first been diagnosed with cherry eye as puppies or juveniles before one year of age (OR = 28.63 [23.13–35.44] compared to young adults).

**Figure 1:**
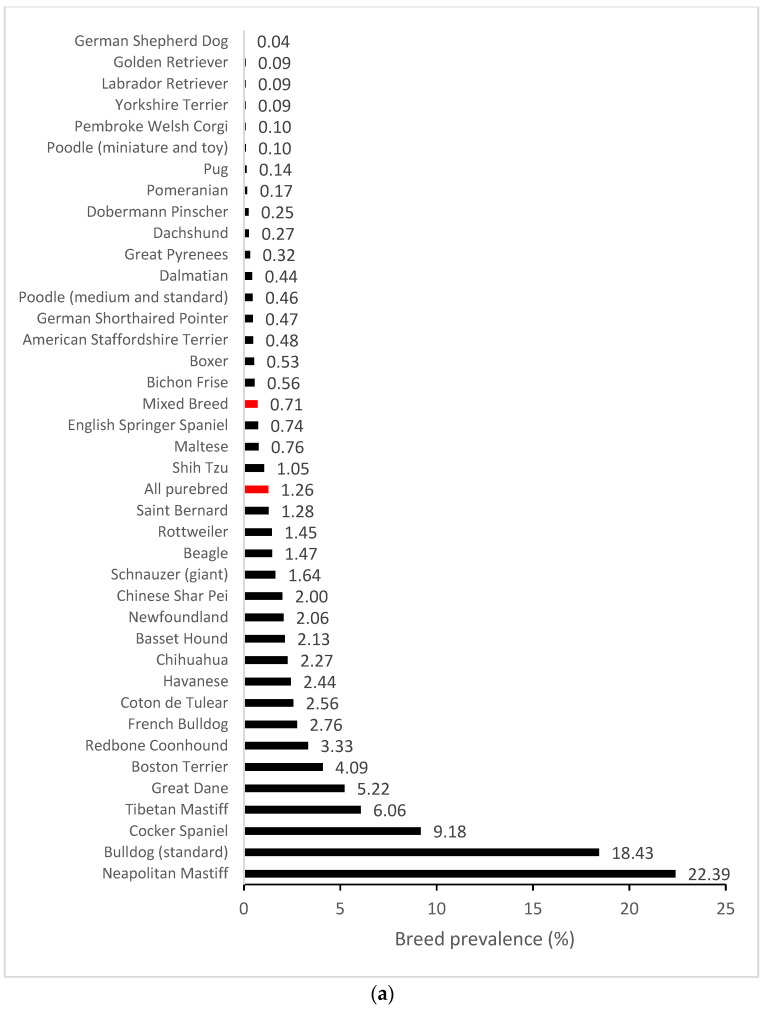

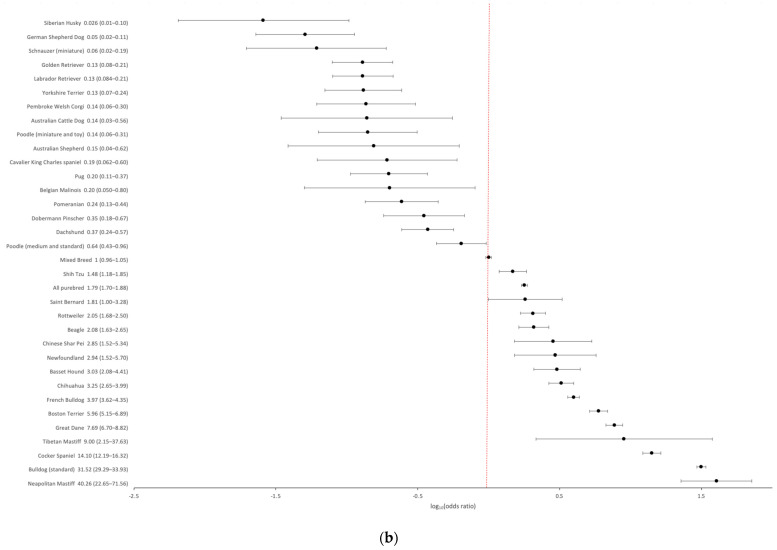
Breed-specific demographics of cherry eye in a US primary veterinary care cohort. (**a**) Breed prevalence in breeds with at least five cases observed and/or an observed prevalence > 2%; (**b**) Breed-specific odds ratios (black circles with 95% confidence interval error bars) for breeds with statistically significant deviations (*p* < 0.05) from the odds in mixed breed dogs. The baseline odds ratio in mixed breed dogs is depicted by the red vertical dotted line.

We further compiled breed-specific cherry eye prevalence statistics and calculated ORs for mixed breed dogs and individual breeds, with at least 30 dogs represented in the EMRs and at least one case of cherry eye observed (*n* = 63) (Supplemental Table S1). The highest prevalence (>5%) of cherry eye was found in Neapolitan Mastiff (22.39%), Standard Bulldog (18.43%), Cocker Spaniel (9.18%), Tibetan Mastiff (6.06%) and Great Dane (5.22%) (Figure 1a). Breed-specific ORs were expressed using the odds in mixed-breed dogs as a reference. Several breeds were highlighted as having greater odds and others as having reduced odds of disease compared to the baseline (Figure 1b).

### 3.2. Genome-Wide Association Analysis

**Figure 2:**
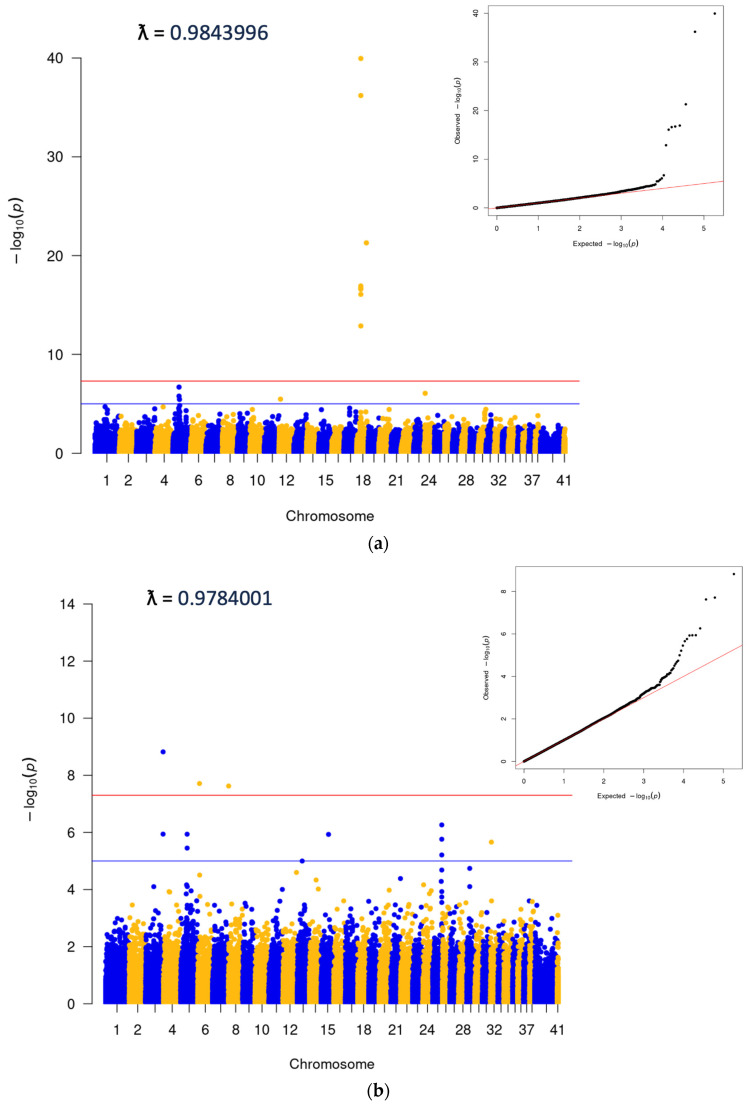

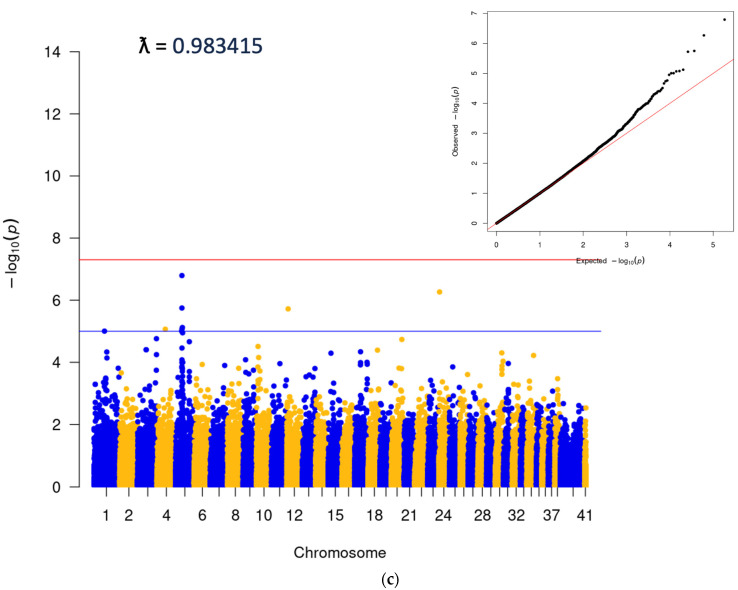
(**a**) Initial clustered GWAS run: 7278 cases and 7278 clustered controls. (**b**) Dogs without copies of *FGF4L1*: 4510 cases and 5119 controls, selected from initial set in 2a. Additional SNP data available in Supplemental Table S2. (**c**) Analysis conditional on 18:20444999 (*FGF4L1*). A total of 7278 cases and 7278 controls.

A subsequent data pull was carried out to identify a GWAS cohort. We identified 7278 cherry eye cases and 7278 controls for association analysis, which was carried out using the GEMMA software package v0.98.5 [15]. The analysis was performed using genetically matched cases and controls, identified using the PLINK [16] cluster command, which uses pairwise identity-by-state (IBS) distance to define the most closely related case and control pairs from an initial 1:5 set of cases and controls to minimize population stratification. Cluster size was set to two and required one case and one control per cluster. Breed demographics in cases were similar to those mentioned for the epidemiologic study and reflected both disease prevalence and the popularity of the breed. The top five breeds with the highest case numbers included the Standard Bulldog, French Bulldog, Great Dane, Cocker Spaniel, and Boston Terrier. The top SNP in the analysis was on CFA18 (18:20444999; *p* = 1.11E^−40^), and corresponded to the locus of the *FGF4L1* retrogene insertion associated with chondrodysplasia in many breeds [17] (Figure 2a). In order to look for other significant associations as well as to confirm the presence of one single peak on CFA18, two additional analyses were performed. The first excluded any cases or controls that had any copies of the *FGF4L1* retrogene insertion (Figure 2b), and the second was run conditionally on the top SNP from the original matched run (Figure 2c). These supplemental tests did confirm the single CFA18 peak as well as highlight some additional SNPs at a significant level (Figure 2c, Supplemental Table S2). The SNP with the lowest *p*-values was a peak on CFA3 corresponding to the gene *LCORL*, which has been associated with body size in many species, including the dog [18]. The other significant SNP that came up with any consistency in the analyses was on CFA6 and corresponded to the gene *CYP3A26*, a cytochrome P450 enzyme present mainly in the liver.

**Figure 3:**
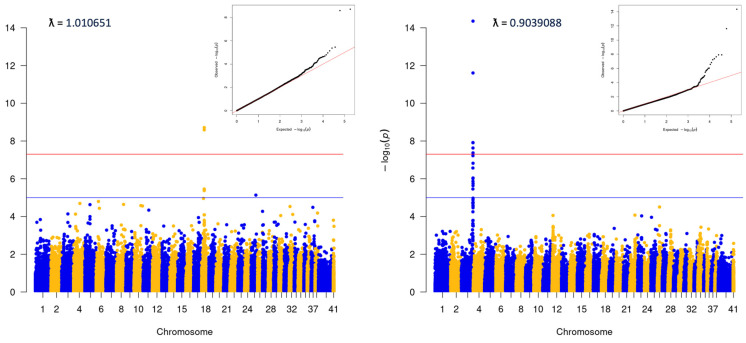
Poodle mix analyses, 40% or greater Poodle ancestry in both cases and controls. L: “Small” (mixes that included Miniature/Toy Poodle), 213 cases and 431 controls. R: “Large” (mixed dogs with ancestry from Standard/Medium Poodles), 120 cases and 130 controls.

**Figure 4:**
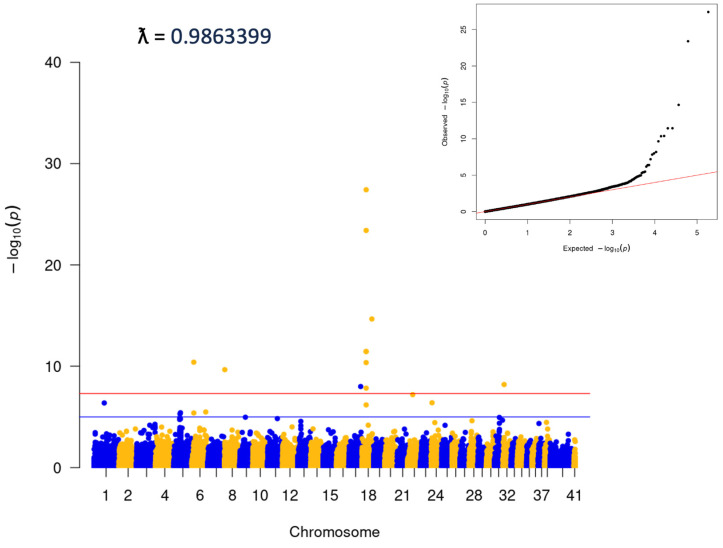
Mixed breed analysis, no dogs with >25% single origin ancestry. A total of 5136 cases and 5236 controls.

Visual analysis of PCA plots indicated little evidence of distinct population stratification (Supplemental Figure S1). Within-breed analyses were run for any breed with ≥100 cases (Supplemental Figure S2). Analysis was also completed for the ‘Puggle’, a common mix including Beagle and Pug. This latter group was comprised dogs with ≥80% ancestry that could be attributed to a combination of the Beagle and Pug breeds. No statistically significant variants were identified in any of the within-breed analyses. Analyses were also performed for two groups of ‘Doodles’: a ‘Large’ group, which included ≥40% ancestry from the Medium/Standard Poodle type, and a ‘Small’ group, which contained ≥40% Medium/Toy Poodle (Figure 3). Both analyses showed significant associations (Large: 3:91269525, *p* = 4.42E^−15^, locus corresponds to the location of the gene *LCORL*, and Small: *p* = 2.56E^−09^, consistent with the *FGF4L1* insertion as above). Finally, an analysis including only dogs classified as mixed-breed was carried out. The dogs in this group did have any more than 25% single-origin ancestry. This analysis was consistent with the initial matched all-breed run (18:20444999; *p* = 3.81E^−28^, Figure 4).

### 3.3. Association between Cherry Eye and the FGF4L1 Retrogene Variant in Mixed Breed Dogs

As a follow-up to the GWAS findings implicating the *FGF4L1* retrogene locus, we leveraged the fact that our custom genotyping array includes a direct assay for the retrogene variant. This assay mapping to 18:20443728 is also featured in the GWAS results as one of the most significant signals. We identified 767,862 dogs (6729 cherry eye cases and 761,133 controls) with both EMR data and genotype data available for *FGF4L1*. The *FGF4L1*-derived allele was significantly associated with the cherry eye phenotype in mixed-breed dogs and in the combined dataset, where the association is likely driven by the larger overall number of mixed-breed dogs compared to purebreds (Table 2). In contrast, the derived allele was significantly underrepresented in cases in the purebred cohort.

### 3.4. Within Breed FGF4L1 Derived Allele Frequency and Clinical Prevalence of Cherry Eye

**Figure 5:**
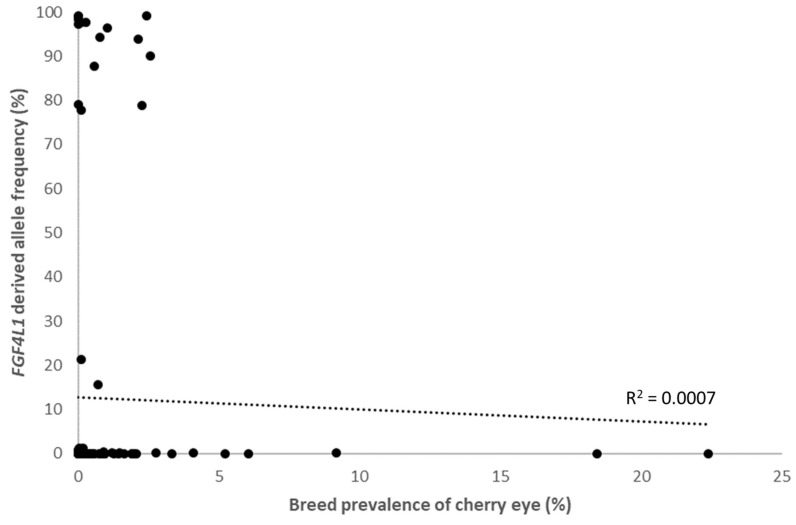
Correlation between *FGF4L1* derived allele frequency and clinical cherry eye prevalence in 115 breeds (black circles).

We plotted the *FGF4L1*-derived allele frequency against clinical cherry eye prevalence in the 115 breeds that had a minimum of 30 dogs with EMR data available. The highest prevalence of cherry eye was found in the Standard Bulldog, Cocker Spaniel and Boston Terrier, which had low *FGF4L1* frequencies (0.006–0.07%). This suggests other clinical and genetic risk factors for cherry eye in these breeds. When grouping by breed populations and comparing across breeds, *FGF4L1* frequency is not correlated with the high prevalence of cherry eye (Spearman correlation coefficient = 0.192, *p* = 0.04; Figure 5).

## 4. Discussion

The introduction of routine genetic panel screening into a veterinary clinical setting enables the pairing of information from EMRs with comprehensive genotype information. We used this powerful approach to ascertain the largest cohort examined for cherry eye to date and explore the genetic basis of the phenotype through a large-scale multi-breed genome-wide association study (GWAS).

We first assessed basic epidemiological parameters such as disease prevalence and basic demographic risk factors. Our observations in an extensive North American primary veterinary care setting largely replicate previous findings from primary care veterinary practices in the United Kingdom (UK) [4]. We observe a cherry eye lifetime prevalence of 0.88%, compared to the previously reported annual prevalence of 0.2% in the UK, and confirm that most diagnoses of cherry eye are made in the first year of a dog’s life. We further replicate the discoveries that purebreds, and particularly brachycephalic breeds, are at higher risk of developing cherry eye. On the breed-specific level, our findings highlight many of the same breeds as in the aforementioned UK study [4] as being at higher and lower disease risk, respectively, compared to mixed-breed dogs. Both studies consistently highlight, e.g., the Neapolitan Mastiff, English/Standard Bulldog and Cocker Spaniel as breeds at highest risk.

GWAS findings showed a consistent peak for all-breed and mixed-breed analyses at the location of the CFA18 *FGF4* retrogene insertion *FGF4L1*. Within-breed analyses were not sufficiently powerful to highlight any variants of genome-wide significance. A closer look at the *FGF4L1*-derived allele association in the full EMR dataset revealed that it was driven by mixed-breed dogs, as suggested by the GWAS findings. While breed structure can be of great benefit for mapping disease variants, in this case, the high divergence of *FGF4L1* frequencies between breeds, from near-fixed to non-existent, complicates the interpretation of the effect of the variant. We also show that there is a limited correlation between *FGF4L1*-derived allele frequencies and the clinical prevalence of cherry eye across breeds, further emphasizing that there are additional breed-specific risk-modifying effects at play.

Fibroblast growth factors, or FGFs, are a group of cell-signaling proteins that are responsible for a vast number of mechanisms in the body. They can affect the differentiation of multiple cell types, including endothelial cells, smooth muscle cells, adipocytes, melanocytes, neurons, and chondrocytes [19]. They play roles in apoptosis, in embryology and stem cell pluripotency, and in cellular proliferation, survival, metabolism, morphogenesis, and differentiation, as well as in angiogenesis and tissue repair and regeneration [20].

In the dog, the *FGF4L1* retrogene has been shown to be the cause of a breed-defining chondrodysplasia [17], which develops as a result of abnormal endochondral ossification [21] and stunted, often asynchronous, growth of the long bones in the limbs, particularly the forelimbs [22]. An additional *FGF4* retrogene insertion on CFA12 was also more recently associated with an increased risk for intervertebral disc disease [23]. *FGF4L1* is thought to possibly have an effect on this phenotype as well, but it remains under study. In humans, the most common genetic cause of achondroplasia (dwarfism) is caused by a substitution mutation in the FGF receptor FGFR3, one of the receptors for FGF4 [24]. Also in humans, constitutional increases in *FGF3* and *FGF4* have been associated with an increased risk of craniosynostosis [25], and *FGF4* has been shown to be a negative prognostic indicator for a number of cancers due to its effect on tumor growth, angiogenesis, invasion and metastasis [26].

The *FGF4* family of retrogenes can induce the expression of Sprouty genes [27], which interferes with the degradation of FGF receptors, essentially causing overexpression of fibroblast growth factors [28]. In the chick, induced overexpression of Sprouty genes has been associated with a reduction in limb bud outgrowth and a reduction in skeletal length resulting from inhibited chondrocyte differentiation [27].

In addition to the roles mentioned above, FGFs are involved in the synthesis of the extracellular matrix, creating connective tissue by placing its components (collagens, proteoglycans, laminins, fibronectin, elastin, and microfibrillar proteins) [29]. They are also involved in active remodeling of the microstructure of the extracellular membrane, as well as affecting its mechanical forces and polarization [29]. In theory, it is reasonable to think that overexpression of an FGF could cause a weakness or laxity in the connective tissue structure, allowing the gland of the nictitans to then prolapse. However, as no follow-up studies have yet been run, this is merely a possible hypothesis for the mechanism of *FGF4L1* as it relates to cherry eye. Further study recommendations may include gene expression studies in relevant tissues to confirm *FGF4L1* expression levels. It could also be interesting to compare the tensile strength of the connective tissue within the nictitans in dogs with and without glandular prolapse in order to see whether the current hypothesis regarding the etiology of cherry eye is upheld. A review of the cartilage shape and histological makeup similar to that performed in [2] may also be enlightening.

Limitations of the current study include possible misinterpretation or misdiagnosis of the study ailment, population stratification, and low purebred case numbers for some specific breeds. Regarding the diagnosis of a prolapsed nictitans, this is normally something that a general practice veterinarian is comfortable diagnosing based on signaling and examination. This, along with the use of a single electronic medical record ailment code for ‘Third Eyelid Gland Prolapse’, greatly reduces the chance that cases were included erroneously. The number of cases included in the study also decreases the effect that any such cases would have on the overall analysis.

While population stratification is always a concern when dealing with differences between dog breeds, the matching of cases and controls was performed in order to decrease the likelihood of this issue. While we first set out to investigate a combined sample set consisting of both purebreds and mixed breed dogs in an unbiased manner, our GWAS discovery implicating *FGF4L1* (a variant responsible for a breed-defining trait that is fixed or near-fixed in many breeds) prompted us to focus on elucidating its association with cherry eye in mixed breed dogs. Finally, PCA plots also indicate that there is little distinct difference between cases and controls.

Though there were a few breeds with markedly high case numbers (Standard Bulldog, *n* = 1238; French Bulldog, *n* = 668), none of the purebred analyses had sufficient power to identify any significant loci. This indicates either that additional cases for each breed would be needed to power identification of cherry eye-associated loci or potentially that risk factors may be fixed within certain breeds. It is likely that there are many gene variants, including protective ones, that contribute to the manifestation of cherry eye, and such genes may differ between breeds. This is exemplified by the lack of an evident correlation between high derived allele frequencies of *FGF4L1* and the high clinical prevalence of cherry eye when comparing across breeds. This importantly emphasizes that *FGF4L1* is a risk factor rather than highly penetrant or causal, that other genetic factors may mitigate the increase in risk entirely, and that other unrelated genetic and clinical factors are likely to cause a high prevalence of cherry eye in some breeds.

In this study, large-scale, mixed-breed analyses have been used to identify a significant association between cherry eye and *FGF4L1*. The study demonstrates the multifactorial nature of this complex disease and shows how the etiology varies between different breeds and mixed-breed dogs. Finally, but importantly, it highlights that the results of large genetic studies using diverse populations may not be applicable to closed population subgroups, such as breeds.

## Figures and Tables

**Table 1:** Demographics of cherry eye in a US primary veterinary care cohort of 769,337 dogs.

Variable	Case Population Characteristics	Odds Ratio (95% CI)	*p*-Value
Sex			
Female	2942 (43.57%)	1	
Male	3811 (56.43%)	1.10 (1.05–1.15)	<0.001
Neuter			
Intact	2773 (41.06%)	1	
Neutered/spayed	3980 (58.94%)	0.79 (0.76–0.83)	<10^−16^
Breed			
Mixed Breed	3826 (56.66%)	1	
Purebred	2927 (43.34%)	1.79 (1.70–1.88)	<0.0001
Skull shape			
Mesocephalic	522 (7.73%)	1	
Brachycephalic	2072 (30.68%)	7.60 (6.90–8.36)	<10^−16^
Dolichocephalic	332 (4.92%)	1.52 (1.32–1.74)	<10^−8^
Uncategorized (mixed breed)	3827 (56.67%)	1.61 (1.47–1.77)	<10^−16^
Age at first diagnosis			
Average	0.83 years		
Median	0.48 years		
Range	0.07–15.36 years		
0–1 years	4743 (79.31%)	28.63 (23.13–35.44)	<10^−16^
1–2 years	774 (12.94%)	4.87 (3.90–6.08)	<10^−16^
2–3 years	252 (4.21%)	1.97 (1.54–2.52)	<10^−7^
3–4 years	86 (1.44%)	1	
4–5 years	39 (0.65%)	2.33 (1.60–3.41)	<0.0001
>5 years	86 (1.44%)	4.34 (3.22–5.85)	<10^−16^

**Table 2:** Association analysis results for the *FGF4L1* retrogene variant. Sample numbers and percentages are shown for genotype groups named by the number of copies (0, 1, 2) of the derived allele known to cause short-leggedness. Odds ratios are shown for dogs with 1 or 2 copies of *FGF4L1*, respectively, in comparison to dogs with 0 copies.

Group	0	1	Odds Ratio (95% CI)	2	Odds Ratio (95% CI)	Total	Chi Square *p*-Value
All							
Cases Controls	4445 (66.06%) 565,269 (74.27%)	753 (11.19%) 83,819 (11.01%)	1.14 (1.06–1.23)	1531 (22.75%) 112,045 (14.72%)	1.74 (1.64–1.84)	6729 761,133	<10^−16^
Purebred							
Cases Controls	2659 (91.00%) 184,865 (81.09%)	39 (1.33%) 8209 (3.60%)	0.33 (0.24–0.45)	224 (7.67%) 34,915 (15.31%)	0.45 (0.39–0.51)	2922 227,989	<10^−16^
Mixed Breed							
Cases Controls	1786 (46.91%) 380,404 (71.35%)	714 (18.75%) 75,610 (14.18%)	2.01 (1.84–2.19)	1307 (34.33%) 77,130 (14.47%)	3.61 (3.36–3.88)	3807 533,144	<10^−16^

## Data Availability

The data presented in this study are openly available in Dryad at https://doi.org/10.5061/dryad.k6djh9wdz.

## Supplementary Materials

The following supporting information can be downloaded at: https://www.mdpi.com/article/10.3390/genes15020198/s1, Figure S1: PCA plots for matched cherry eye GWAS; Figure S2: Within-breed analyses; Table S1: Cherry eye prevalence and ORs by breed group; Table S2: Significant SNPs at other loci.
