# Supplementary material for: Association of FGF4L1 Retrogene Insertion with Prolapsed Gland of the Nictitans (Cherry Eye) in Dogs
Source: Genes (Basel). 2024 Feb 1;15(2):198. doi: 10.3390/genes15020198 (PMC10887708; doi:10.3390/genes15020198)

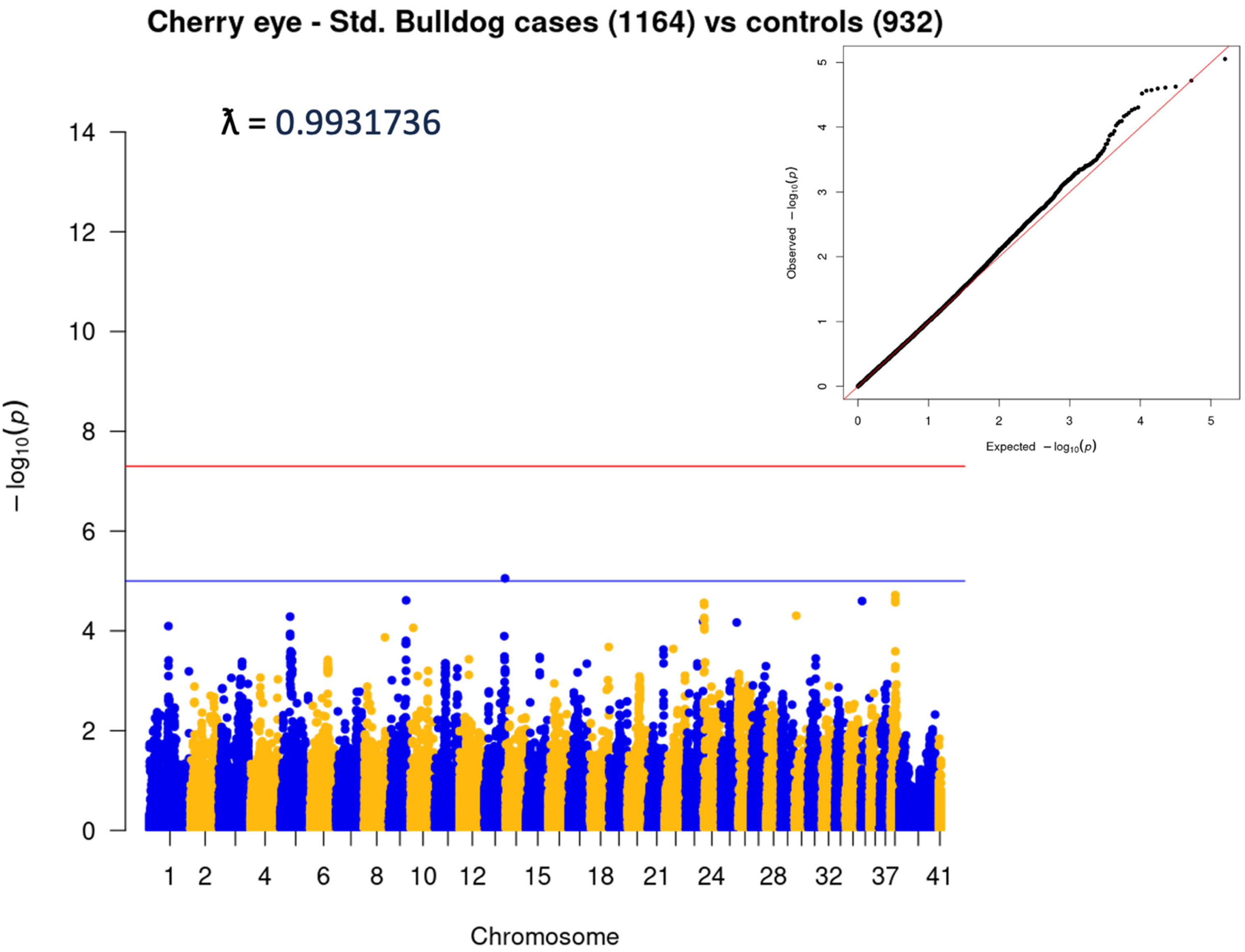

Cherry eye - Std. Bulldog cases (1164) vs controls (932)
λ = 0.9931736
14
12
10
8
6
4
2
0
− log10(p)
1
2
4
6
8
10
12
15
18
21
24
28
32
37
41
Chromosome
Observed − log10(p)
Expected − log10(p)
0
1
2
3
4
5

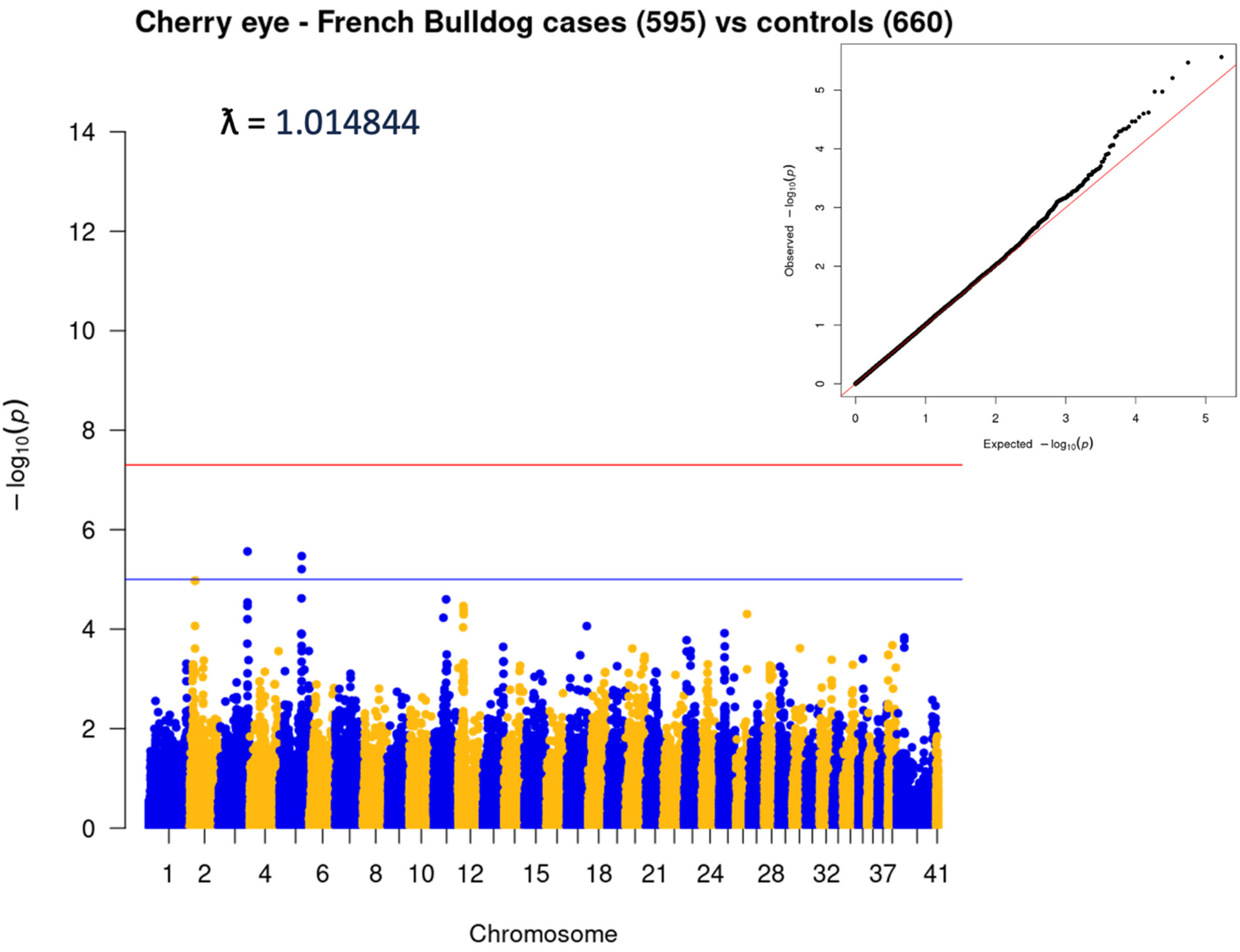

Cherry eye - French Bulldog cases (595) vs controls (660)
λ = 1.014844
14
12
10
8
6
4
2
0
$-\log_{10}(p)$
1 2 4 6 8 10 12 15 18 21 24 28 32 37 41
Chromosome
Observed $-\log_{10}(p)$
Expected $-\log_{10}(p)$
0 1 2 3 4 5

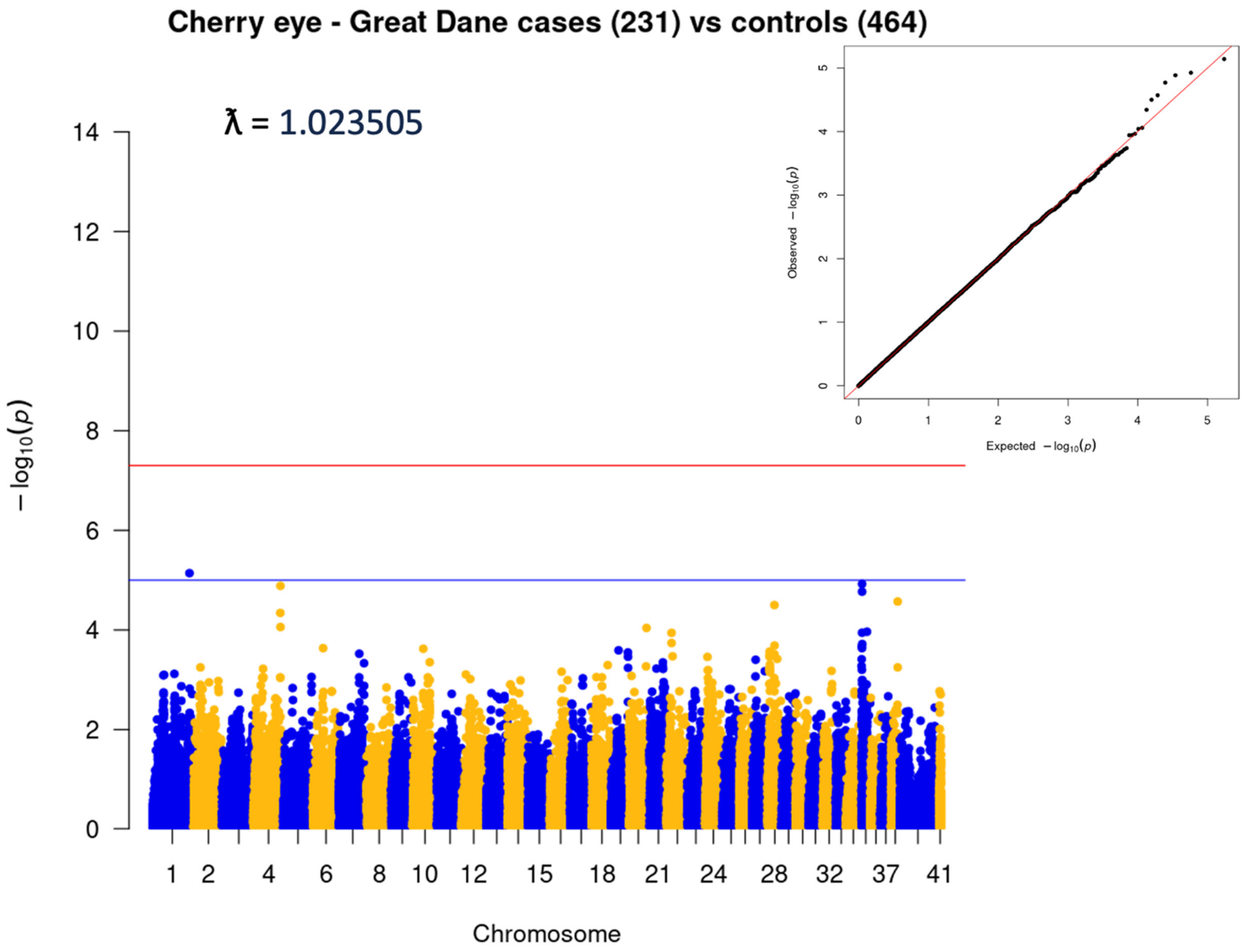
Cherry eye - Great Dane cases (231) vs controls (464)
λ = 1.023505
−log10(p)
Chromosome
Observed −log10(p)
Expected −log10(p)

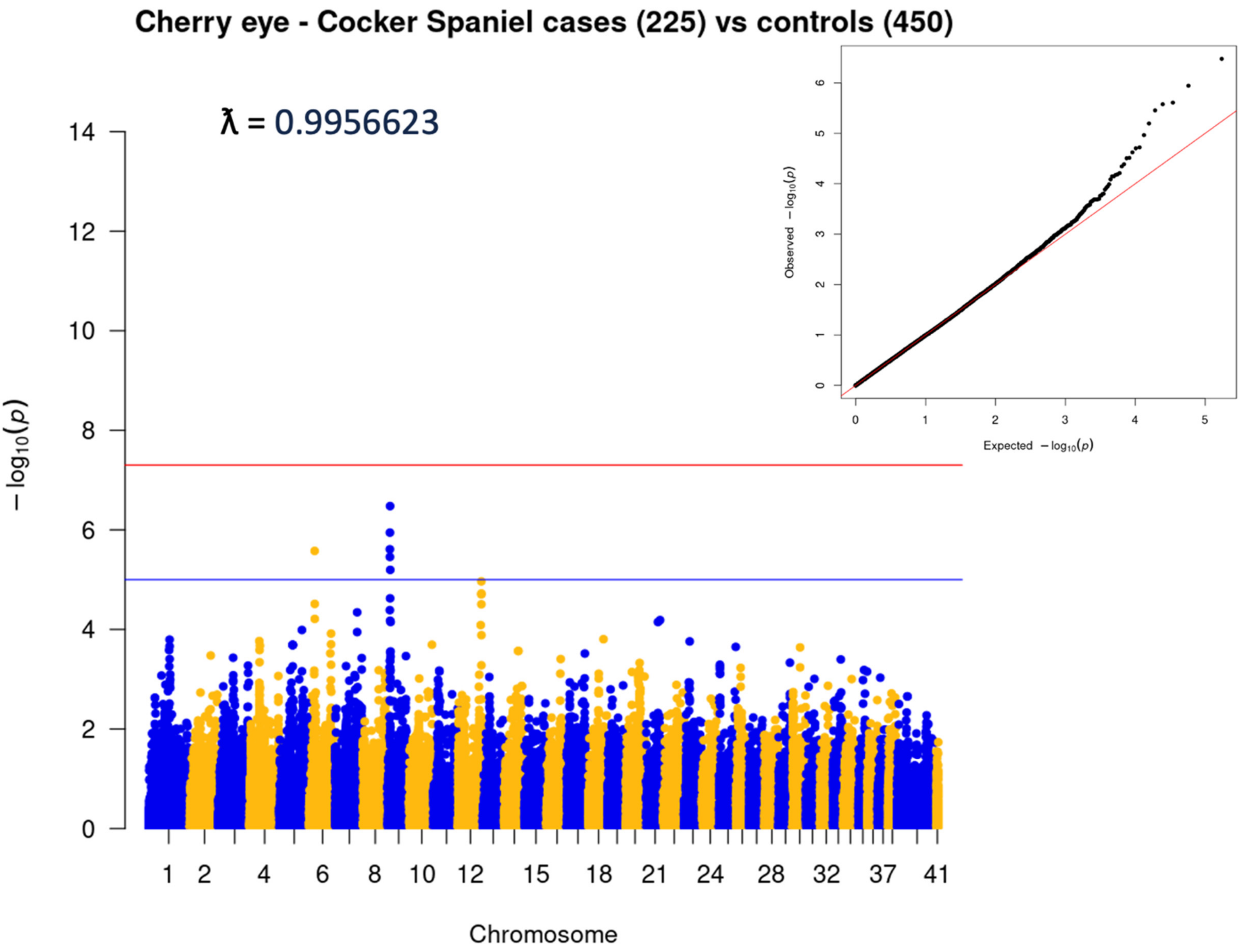

Cherry eye - Cocker Spaniel cases (225) vs controls (450)
λ = 0.9956623
14
12
10
8
6
4
2
0
−log10(p)
1 2 4 6 8 10 12 15 18 21 24 28 32 37 41
Chromosome
Observed −log10(p)
Expected −log10(p)

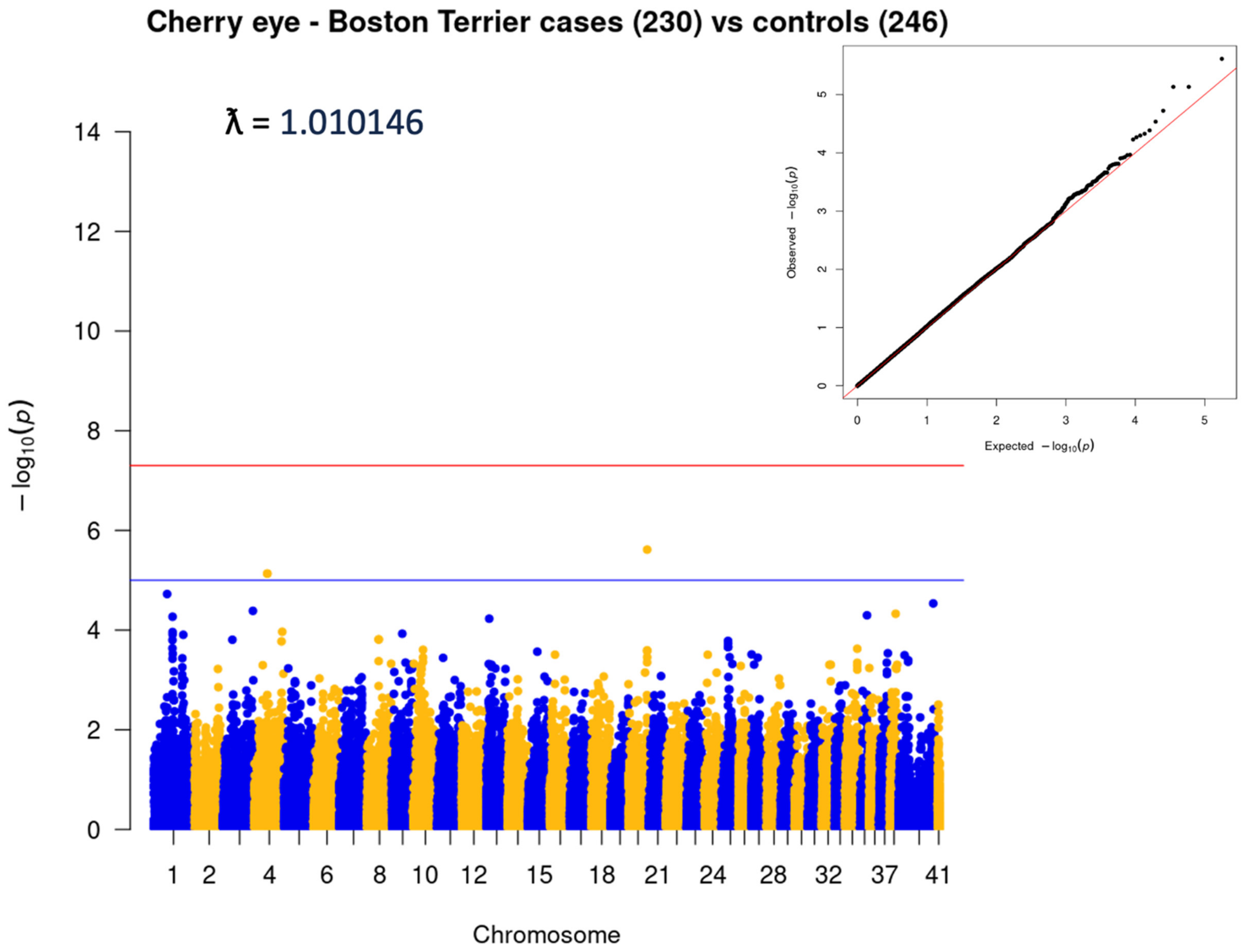
Cherry eye - Boston Terrier cases (230) vs controls (246)
λ = 1.010146
$-\log_{10}(p)$
Chromosome
Observed $-\log_{10}(p)$
Expected $-\log_{10}(p)$

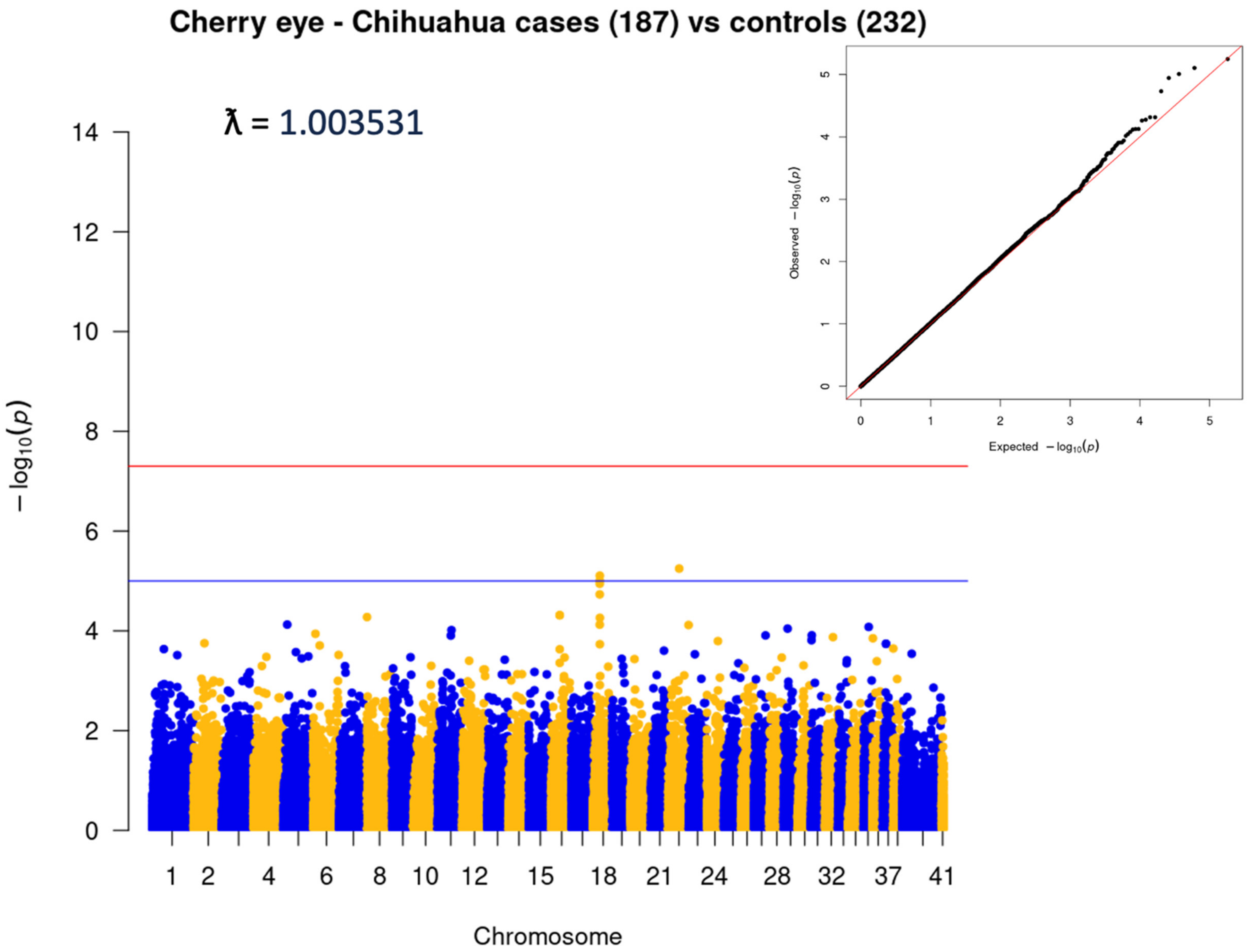

Cherry eye - Chihuahua cases (187) vs controls (232)
λ = 1.003531
$-\log_{10}(p)$
Chromosome
1 2 4 6 8 10 12 15 18 21 24 28 32 37 41
Observed $-\log_{10}(p)$
Expected $-\log_{10}(p)$

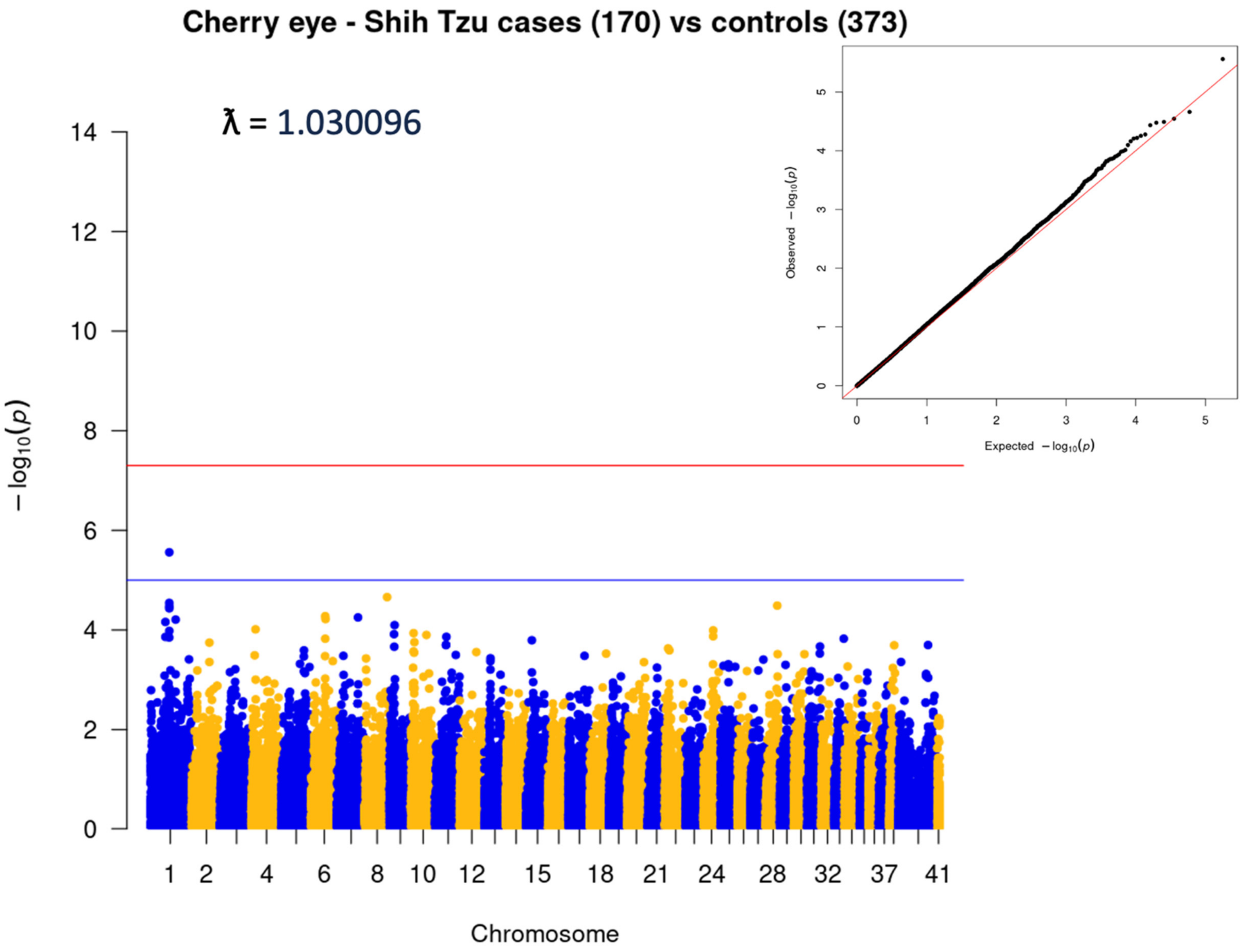
Cherry eye - Shih Tzu cases (170) vs controls (373)
λ = 1.030096
14
12
10
8
6
4
2
0
$-\log_{10}(p)$
1
2
4
6
8
10
12
15
18
21
24
28
32
37
41
Chromosome
Observed $-\log_{10}(p)$
Expected $-\log_{10}(p)$
0
1
2
3
4
5

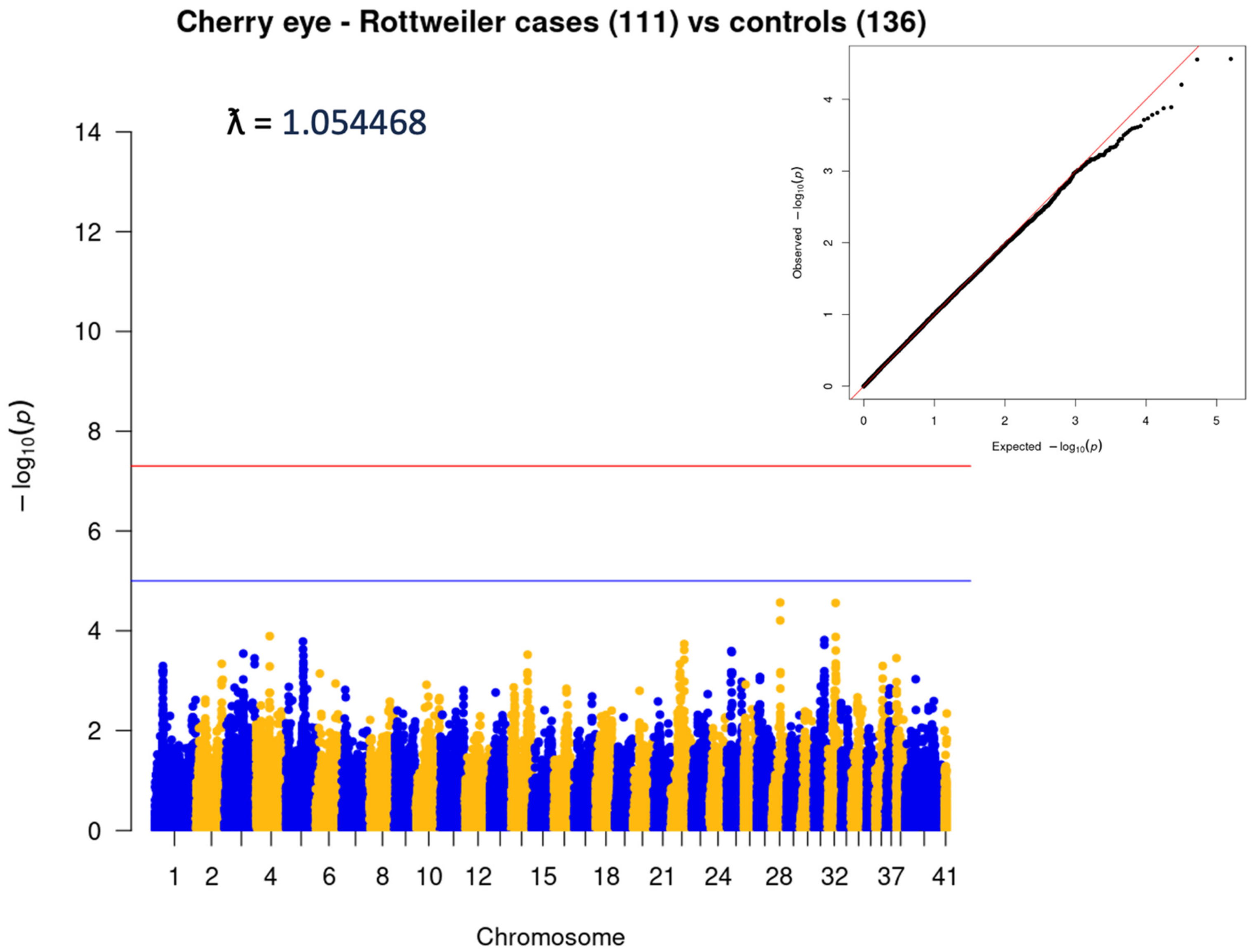

Cherry eye - Rottweiler cases (111) vs controls (136)
λ = 1.054468
$-\log_{10}(p)$
14
12
10
8
6
4
2
0
1 2 4 6 8 10 12 15 18 21 24 28 32 37 41
Chromosome
Observed $-\log_{10}(p)$
Expected $-\log_{10}(p)$

Cherry eye - Puggle cases (114) vs. controls (177)

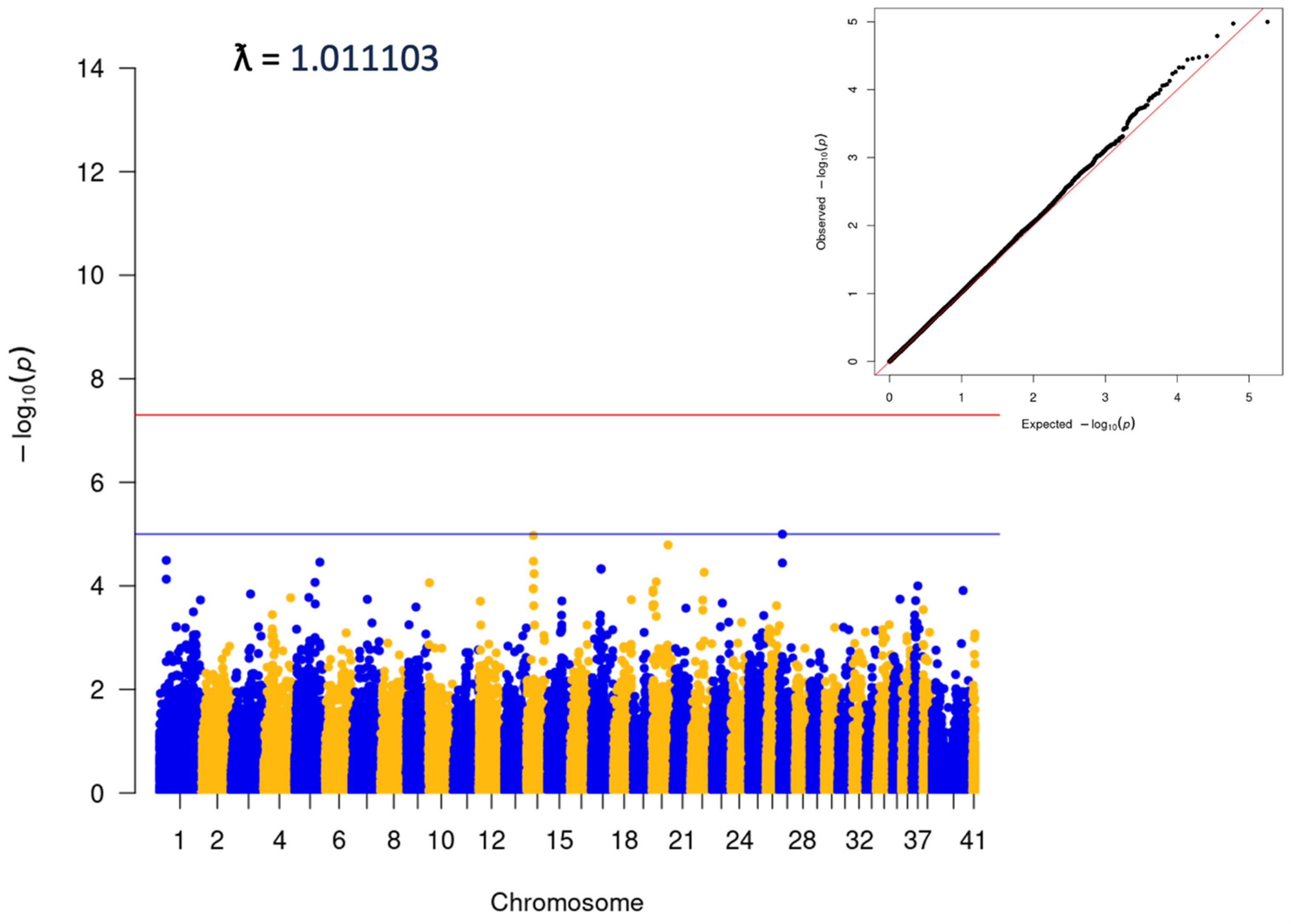

Supplement: Supplementary file 1 [file genes-15-00198-s001.zip › Cherry_eye_Supp_Figure_S2.pdf]
