# Supplementary material for: Association of FGF4L1 Retrogene Insertion with Prolapsed Gland of the Nictitans (Cherry Eye) in Dogs
Source: Genes (Basel). 2024 Feb 1;15(2):198. doi: 10.3390/genes15020198 (PMC10887708; doi:10.3390/genes15020198)
Supplement: Supplementary file 1 [file genes-15-00198-s001.zip › Cherry_eye_Supp_table_S2.pdf]

GWAS Analysis	Chromosome	SNP	P-value
Doodle (Large)	2942 (43.57%)	1	
	3811 (56.43%)	1.10 (1.05-1.15)	< 0.001
Doodle (Small)	2773 (41.06%)	1	
	3980 (58.94%)	0.79 (0.76-0.83)	< 10^-16
No *FGF4L1* dogs	3826 (56.66%)	1	
	2927 (43.34%)	1.79 (1.70-1.88)	< 0.0001
Mixed breed only	522 (7.73%)	1	
	2072 (30.68%)	7.60 (6.90-8.36)	< 10^-16
	332 (4.92%)	1.52 (1.32-1.74)	< 10^-8
	3827 (56.67%)	1.61 (1.47-1.77)	< 10^-16

GWAS Analysis	Chromosome	SNP	P-value	Gene
No *FGF4L1* dogs	3	3:91269525	1.51E-09	*LCORL*
	6	6:9777717	1.94E-08	*CYP3A26*
	8	8:880421	2.38E-08	
Doodle (Large)	3	3:91269525	4.42E-15	*LCORL*
Doodle (Small)	18	18:20444999	2.56E-09	*FGF4L1*
Mixed breed only	6	6:9777717	4.07E-11	*CYP3A26*
	8	8:880421	2.22E-10	
	17	17:57776247	1.02E-08	*CHD1L*
	22	22:24394293	6.42E-08	*KLHL1*
	32	32:4842721	6.48E-09	*C32H4orf22*
