# Supplementary figures and images for: Association of FGF4L1 Retrogene Insertion with Prolapsed Gland of the Nictitans (Cherry Eye) in Dogs
Source: Genes (Basel). 2024 Feb 1;15(2):198. doi: 10.3390/genes15020198 (PMC10887708; doi:10.3390/genes15020198)

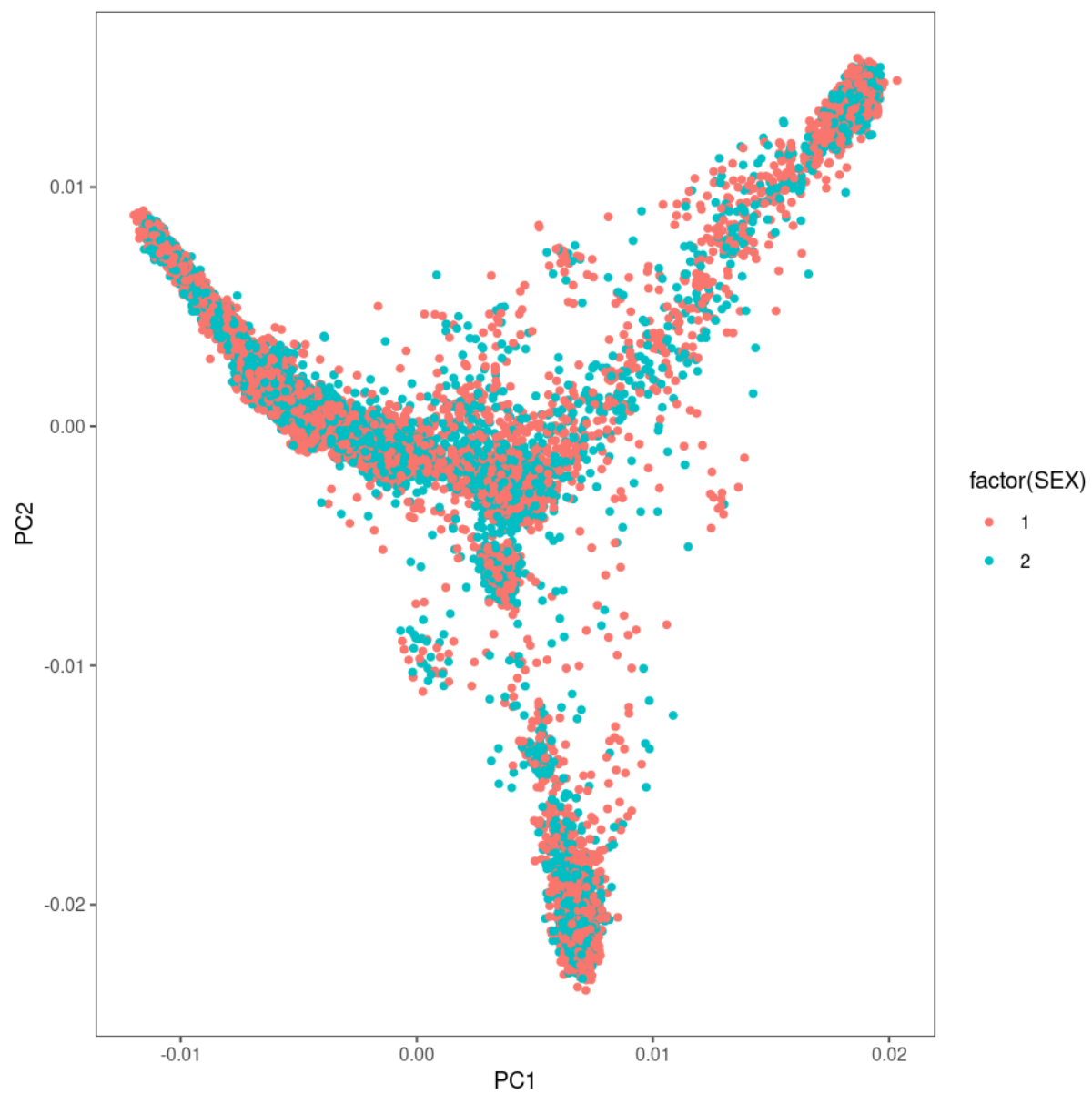

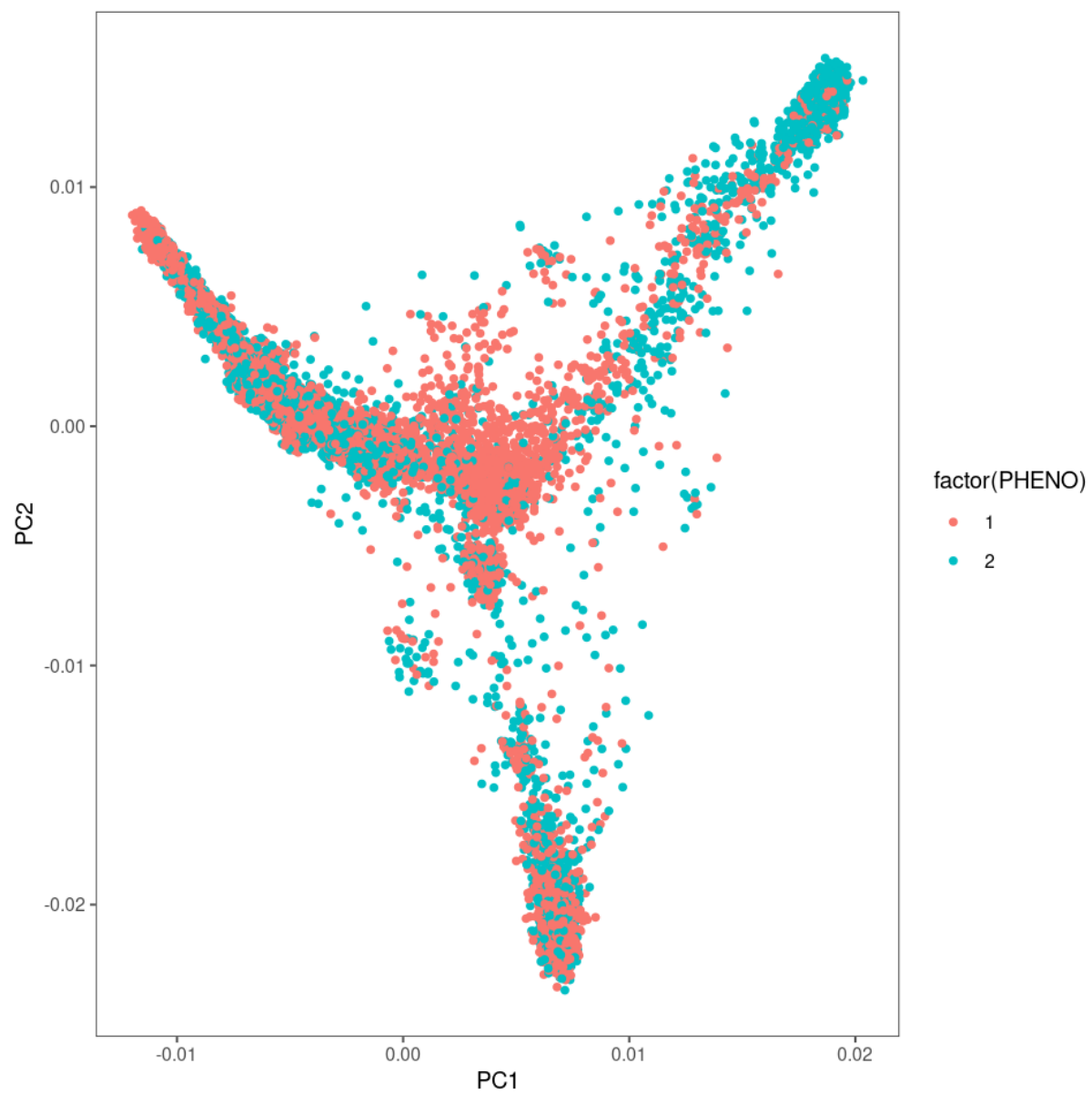

**Supplemental Figure S1.** PCA plots by gender (1 = male, 2 = female) and phenotype (control = 1, case = 2).

Supplement: Supplementary file 1 [file genes-15-00198-s001.zip › Cherry_eye_Supp_Figure_S1.pdf]
